# Reusable and Practical Biocomposite Based on *Sphingopyxis* sp. YF1 and Polyacrylonitrile-Based Carbon Fiber for the Efficient Bioremediation of Microcystin-LR-Contaminated Water

**DOI:** 10.3390/toxins16010020

**Published:** 2023-12-29

**Authors:** Tian Ma, Jiajia Zhang, Lili Yang, Shengyu Zhang, Xizi Long, Qingyi Zeng, Zhongyu Li, Xiaoya Ren, Fei Yang

**Affiliations:** 1 Hunan Province Key Laboratory of Typical Environmental Pollution and Health Hazards, School of Public Health, University of South China, Hengyang 421001, China; mt15075392929@163.com (T.M.);; 2 Hunan Provincial Key Laboratory of Clinical Epidemiology, Xiang Ya School of Public Health, Central South University, Changsha 410078, China; 3 School of Resources & Environment and Safety Engineering, University of South China, Hengyang 421001, China; 4 Institute of Pathogenic Biology, School of Nursing, Hengyang Medical College, Hunan Provincial Key Laboratory for Special Pathogens Prevention and Control, University of South China, Hengyang 421001, China

**Keywords:** microcystin-LR, microbial immobilization, biodegradation, *Sphingopyxis* sp. YF1

## Abstract

Microbial degradation is a cost-effective and environmentally friendly method for removing microcystin-LR (MC-LR). However, the application of free bacteria has limitations due to low operational stability and difficulties in recovery. In a previous study, our group successfully isolated a highly efficient MC-LR-degrading bacterium, *Sphingopyxis* sp. YF1, from Taihu. To enhance its practical potential in addressing MC-LR-contaminated water pollution, a novel biological material named polyacrylonitrile-based carbon fiber @*Sphingopyxis* sp. YF1 (PAN-CF@YF1) was synthesized. The immobilization conditions of strain *Sphingopyxis* sp. YF1 on PAN-CF surfaces were optimized using Box–Behnken design and response surface methodology (RSM), which turned out to be an optimal pH of 7.6 for the culture medium, a ratio of 0.038 g of supporting materials per 100 mL of culture media, and an incubation time of 53.4 h. The resultant PAN-CF@YF1 showed a great degradation effect both for low and high concentrations of MC-LR and exhibited satisfactory cyclic stability (85.75% after six cycles). Moreover, the application of PAN-CF@YF1 in the bioreactors demonstrated effective and sustainable MC-LR removal, with a removal efficiency of 78.83% after three consecutive treatments. Therefore, PAN-CF@YF1 with high degradation activity, environmental compatibility, straightforward preparation, and recyclability shows significant application potential for the bioremediation of MC-LR-contaminated water bodies.

## 1. Introduction

With intensive eutrophication and climate change, harmful algal blooms (HABs) have become increasingly prevalent in freshwater globally [1,2]. In addition to the unpleasant tastes and odors caused by HABs, some cyanobacteria can generate toxic microcystins (MCs) that, when released into the aquatic environment, would pose huge risks to ecosystems and drinking water safety [3,4,5]. The toxicity of MCs has been well reported. Acute exposure to MCs can induce serious damage to the liver, kidney, and digestive and neurological systems, and it can even lead to death. Long-term exposure to MCs would increase the incidence of cancer [6,7,8]. Among over 250 MC variants, microcystin-LR (MC-LR) is frequently detected and highly toxic [9,10]. Due to the existence of the ring structure, MC-LR is very stable and keeps its toxicity despite the impact of a variety of physical and chemical variables, including high temperature or air pressure, extreme pH, and visible light [7,11]. The International Agency for Research on Cancer (IARC) has identified MC-LR as a potential Group 2B carcinogen [12]. Thus, the effective removal of MC-LR from aquatic systems is crucial.

Various physical and chemical approaches have been applied to eliminate MC-LR pollution, including adsorption on activated carbon [13], chlorination [14], photocatalytic degradation [15], potassium permanganate oxidation [16], and so on. However, these techniques are not effectively implemented due to the high treatment costs and potential secondary pollution caused by waste adsorbent or the added chemical agents [16,17,18,19]. Moreover, some techniques would result in the generation of carcinogenic byproducts during the treatment of MC-LR. For instance, although solar photo-Fenton treatment realized complete removal of MC-LR, about 68% inhibition of protein phosphatase-1 remained for the degradation products. More environmentally friendly and economic methods should be developed to control MC-LR pollution.

Microbial degradation is gaining recognition as an effective and eco-friendly method for the removal of MC-LR [20]. However, free bacteria are easily flushed out of the system or consumed by protozoa, which leads to an unsustainable high efficiency of pollutant degradation in the water treatment system [21]. Difficulties in the recovery and reuse of free bacteria also restrict its practical application. Microbial immobilization offers a viable solution to address these issues [22,23]. Previously, Morón-López et al. immobilized *Sphingopyxis* sp. IM-1 on the recycled reverse osmosis membranes and successfully enabled the formation of MC-degrading biofilm [24]. *Sphingopyxis* sp. is a well-studied MC-LR-degrading bacterium that can sequentially cleave the cyclic heptapeptide MC-LR into linear forms, tetrapeptides, Adda, and smaller amino acids through the action of the *mlrA*, *mlrB*, *mlrC*, and *mlrD* genes [20,25]. However, the degradation product Adda still exhibits certain toxicity [25,26]. We isolated a novel bacterium, *Sphingopyxis* sp. YF1, that is capable of degrading MC-LR from Lake Taihu. It not only possesses higher degradation efficiency than other reported bacteria but also achieves complete detoxification of MC-LR [27]. In the degradation pathway of *Sphingopyxis* sp. YF1, Adda is transformed into phenylacetic acid by aminotransferase and beta oxidase, which is further degraded by the phenylacetic acid enzyme and finally mineralized to CO_2_ through the tricarboxylic acid cycle [28]. Therefore, *Sphingopyxis* sp. YF1 shows great potential for the treatment of MC-LR-contaminated water.

Among various strategies for microbial immobilization, adsorption is a commonly used method [29,30]. Adsorption immobilization of *Sphingopyxis* sp. YF1 on the surface of water-insoluble materials shows some superiorities over the method of embedding [31] or physical adsorption [32] applied in our previous study, such as easy operation, well-preserved microbial activity, without the involvement of chemical agents, and being more suitable for large-scale applications. The solid support used in this study is polyacrylonitrile-based carbon fiber (PAN-CF), which is a high-strength, lightweight fiber material prepared from polyacrylonitrile precursor fibers through processes such as wet spinning, stabilization, and carbonization [33]. PAN-CF possesses strong chemical stability, high mechanical strength, flexibility, and good biocompatibility, making it an excellent bio-carrier for real water treatment and large-scale application [34]. Many studies also reported the formation of biofilm on carbon fiber due to the oxygen-containing groups and sufficient surface area [35,36]. Therefore, to keep the superior degradation performance of *Sphingopyxis* sp. YF1 and improve its practical applicability, PAN-CF and *Sphingopyxis* sp. YF1 were coupled for the effective removal of MC-LR.

In this study, a highly practicable and efficient biomaterial was prepared using PAN-CF as the biocarrier and *Sphingopyxis* sp. YF1 as the immobilization strain, which was applied for the degradation of MC-LR, a frequently observed and highly toxic microcystin in eutrophic water. The characterization of the biomaterial was observed using a scanning electron microscope (SEM). The optimal immobilization conditions (pH value, quality of support material, and immobilization time) of PAN-CF@YF1 were analyzed by response surface methodology (RSM) to allow more cells to be immobilized on the material. Further, the degradation capability, reusability, and stability of MC-LR by PAN-CF@YF1 were investigated. Moreover, a packed bed bioreactor (PBBR) was designed to investigate its potential application and performance in addressing MC-LR contamination in water environments, which is rarely studied previously.

## 2. Results and Discussion

### 2.1. Characterization of PAN-CF@YF1

**Figure 1:**
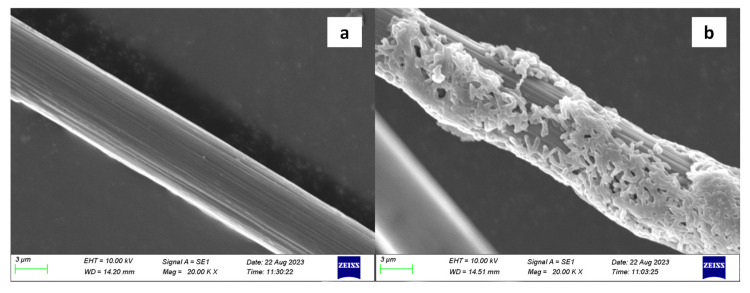
SEM images of PAN-CF (**a**) and PAN-CF@YF1 (**b**).

The selection of carriers is a crucial step in the construction process of bacterial immobilization systems, which requires being environmentally and microorganism-friendly and cost-effective with stable physical and chemical properties [37]. To demonstrate the feasibility of PAN-CF as a microbial carrier for *Sphingopyxis* sp. YF1, we conducted the SEM analysis of PAN-CF before and after immobilization. As shown in Figure 1a, the surface of pristine PAN-CF is rough with varying depths of grooves, while a number of *Sphingopyxis* sp. YF1 cells attached to the surface of PAN-CF are observed after immobilization (Figure 1b), and the bacterial morphology is clear and intact. This indicates the successful preparation of PAN-CF@YF1. One of the indicators of a high-quality carrier is having a large surface area while not causing damage to the microorganisms [38], and carbon fiber has been widely recognized as a suitable biocarrier [39]. In a study comparing four different carrier materials for microbial immobilization, Shinya Mastumoto discovered that carbon fiber exhibited the highest microbial adsorption capacity and fastest adsorption rate among them [40]. In this study, the rough surface of PAN-CF provides an abundant site for microbial attachment. Meanwhile, PAN-CF was non-toxic, as demonstrated in Figure 1b and Figure S1. Therefore, PAN-CF is an excellent carrier for immobilizing bacteria.

### 2.2. The Selection of Immobilization Conditions for Sphingopyxis sp. YF1

#### 2.2.1. Effect of Supporting Materials

**Figure 2:**
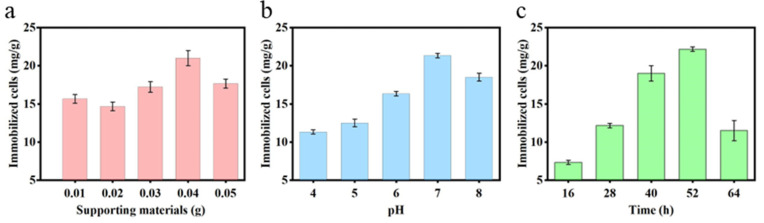
Different immobilization conditions. (**a**) Effect of different supporting material qualities on the immobilization of *Sphingopyxis* sp. YF1 onto PAN-CF (pH = 7.0; time = 48 h). (**b**) Effect of different pH on the immobilization of *Sphingopyxis* sp. YF1 onto PAN-CF (supporting materials = 0.04 g; time = 48 h). (**c**) Effect of immobilization time on the immobilization of *Sphingopyxis* sp. YF1 onto PAN-CF (pH = 7.0; supporting materials = 0.04 g).

The influence of PAN-CF mass on the immobilization of *Sphingopyxis* sp. YF1 is depicted in Figure 2a. Under conditions of pH 7 and a 48 h immobilization duration, the number of *Sphingopyxis* sp. YF1 immobilized by PAN-CF initially decreases, then increases, and subsequently decreases again as the carrier mass increases. When the mass of PAN-CF reached 0.04 g per 100 mL of culture medium, the cell density of *Sphingopyxis* sp. YF1 reached its peak at 21.33 mg/g. However, as the PAN-CF mass increases to 0.05 g per 100 mL of the culture medium, the cell density of *Sphingopyxis* sp. YF1 immobilized on the surface of PAN-CF per gram decreases to 17.67 mg/g. This suggests that the optimal immobilization effect for YF1 is achieved by adding 0.04 g of PAN-CF to 100 mL of the culture medium. Conventionally, increasing the amount of support material with a larger surface area facilitates the immobilization of a greater number of cells [38]. However, this study reveals a contrasting trend. The reason for this discrepancy is the constant volume of the culture medium as the supporting materials increase. Excessive filamentous material fails to adequately extend within the confined environment, resulting in a reduced area between the support material and the medium volume. Consequently, the number of immobilized cells declines. In the study of Nie et al. [41], optimizing immobilization for conditions for the *Pseudomonas fluorescens* strain FF on the surface of waste packaging material polyethylene foam (PEF) using response surface methodology, it was also found that the quality of the carrier material directly affected the immobilization of more bacteria. Optimizing the supporting materials not only improves immobilization efficiency but also helps save costs, aligning with economic benefits.

#### 2.2.2. Effect of pH and Time

The trend depicted in Figure 2b reveals that the quantity of immobilized cells follows an initial increase followed by a decrease as the pH varies within the range of 4–8. Notably, at pH 7, the cell quantity reach peak. indicating a significant influence of pH on the immobilization of *Sphingopyxis* sp. YF1 onto PAN-CF. Previous research has highlighted the preference of *Sphingopyxis* sp. YF1 for neutral conditions [27], suggesting that immobilization of *Sphingopyxis* sp. YF1 on PAN-CF is most effective at pH 7. As shown in Figure 2c, with the prolonged time of immobilizing *Sphingopyxis* sp. YF1 on PAN-CF, at 52 h, the bacterial quantity is at its maximum. This may be attributed to the continuous increase in the optical density (OD) of the bacterial suspension. After reaching the plateau phase, the nutrient levels in the environment decline, causing a decrease in bacterial quantity. Additionally, some bacteria attached to the supporting material may die, leading to a reduction in the immobilized bacterial quantity [42]. Therefore, it is necessary to control the immobilization time.

### 2.3. Box–Behnken Design (BBD) for Sphingopyxis sp. YF1 Immobilization Improvement

On the basis of a single MC-LR degradation experiment, 17 experiments and the interaction of factors such as pH, immobilization time, and supporting materials were used to determine the MC-LR degradation rate. BBD was employed for test optimization. The results of the ANOVA conducted on the BBD experimental data are shown in Table 1. Using Design-Expert version 10 software, the regression findings were fitted to the experimental data to develop a quadratic multiple regression model:

Immobilized cells (mg/g) = 21.75 + 2.73A − 1.86B + 3.20C − 1.00AB + 0.63AC + 2.72BC − 2.59A^2^ − 4.16B^2^ − 3.53C^2^

The F value was the ratio between the mean square of factors in different groups and the mean square of factors in the same group in the ANOVA (Table 1). The model’s F and *p* values were 44.22 and less than 0.0001, suggesting that the regression model was highly significant (*p* < 0.01). The *p* values of the lack of fit were 0.7446, which were not statistically significant, suggesting that the model may be utilized to optimize the immobilization of *Sphingopyxis* sp. YF1 on PAN-CF. According to ANOVA, time had a significant impact on the immobilization of *Sphingopyxis* sp. YF1. The correlation coefficient (R^2^ = 0.9827) revealed that the quadratic multiple regression equation reflected the link between the three parameters and the quantity of immobilized *Sphingopyxis* sp. YF1 more accurately. In addition, a nearly high value of the adjusted determination coefficient (Adjusted R^2^ = 0.9605) demonstrated the model’s significant importance.

**Figure 3:**
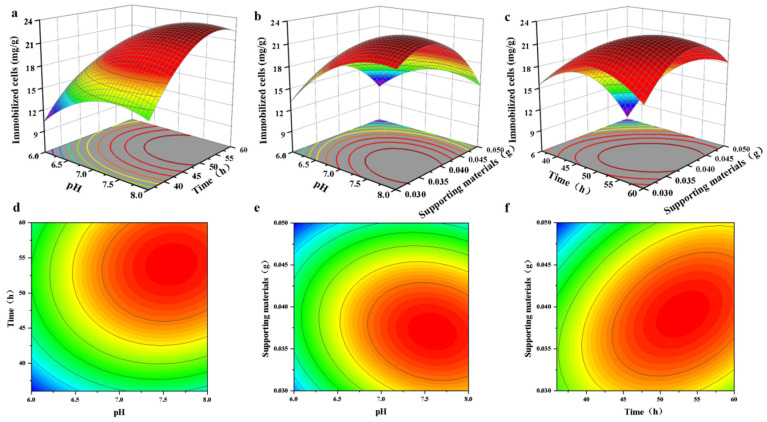
Response surface of environmental factors (**a**) Response surface of pH and immobilization time on the immobilization efficiency. (**b**) Response surface of immobilization time and supporting materials on the immobilization efficiency. (**c**) Response surface of pH and supporting materials on the immobilization efficiency. (**d**) Contour map showing the influence of pH and immobilization time on the immobilization efficiency. (**e**) Contour map showing the influence of immobilization time and supporting materials on the immobilization efficiency. (**f**) Contour map showing the influence of pH and supporting materials on the immobilization efficiency.

As shown in Figure 3, the interaction effects of pH, supporting materials, and immobilization time on the immobilization effectiveness of *Sphingopyxis* sp. YF1 were predicted using a three-dimensional (3D) and two-dimensional (2D) response surface and contour plot, respectively. A detailed analysis of the BBD results is shown in Text S1. The optimal immobilization conditions obtained from the experiments were as follows: pH of 7.6, a ratio of 0.038 g of supporting materials per 100 mL of growth media, and an immobilization time of 53.4 h. Under these conditions, the immobilized cells of *Sphingopyxis* sp. YF1 on surface-supporting materials obtained a value of 23.44 mg (cells)/g(PAN-CF) on average, similar to the predicted value of 23.85 mg (cells)/g(PAN-CF).

### 2.4. Removal of MC-LR by PAN-CF@YF1

**Figure 4:**
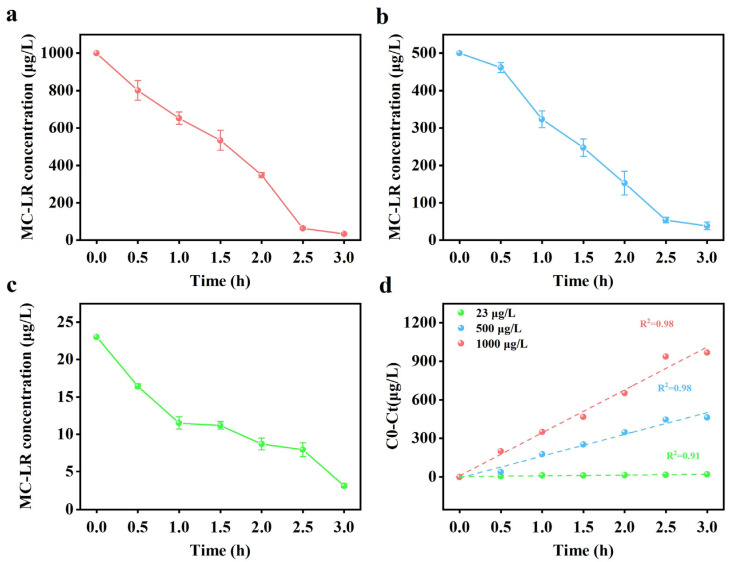
Degradation graph of MC-LR at concentrations of 1000 μg/L (**a**), 500 μg/L (**b**), and 23 μg/L (**c**) by PAN-CF@YF1. Linear plots of zero-order degradation kinetics curve at different initial MC-LR concentrations (**d**).

In previous research, the concentration of MC-LR in water during cyanobacterial blooms ranged from μg/L to mg/L [25,43]. To evaluate the effectiveness of PAN-CF@YF1 under different conditions, we selected three levels of MC-LR concentrations (high, medium, and low) to simulate the water environment during cyanobacterial blooms. Figure 4a–c depicts the time course of MC-LR degradation. When the concentrations of MC-LR were 1000 μg/L, 500 μg/L, and 23 μg/L, effective removal occurred within 3 h with efficiencies of 96.61%, 92.35%, and 86.35%, respectively. Figure 4d represents the study on the degradation kinetics of MC-LR, indicating a zero-order kinetic model. The degradation rate constant increases with the increase in MC-LR concentration. When the initial concentrations of MC-LR were 1000, 500, and 23 μg/L, the reaction rate constants were 333.96, 169.44, and 6.06 μg/L/h, respectively. These findings suggest that as the concentration of MC-LR increases, the removal capacity of PAN-CF@YF1 for MC-LR also increases. Our experimental results are consistent with the study conducted by S.M.K. Nybom et al., where they investigated the removal of MC-LR using Bifidobacterium. They observed a decreasing trend in the removal efficiency of Bifidobacterium as the concentration of MC-LR decreased [44]. This finding aligns with our experimental results and further validates the concentration-dependent removal capacity of PAN-CF@YF1 for MC-LR. The possible reason for this phenomenon could be that high concentrations of MC-LR provide more target substrates, allowing *Sphingopyxis* sp. YF1 on PAN-CF@YF1 to interact more fully, thereby increasing the utilization efficiency of *Sphingopyxis* sp. YF1 for MC-LR and enhancing the degradation rate [45,46]. Table S2 shows the methods used by others to remove MC-LR using free bacteria and immobilized bacteria. These methods exhibit lower degradation efficiency for MC-LR compared to PAN-CF@YF1. In conclusion, the experimental results indicate that PAN-CF@YF1 can effectively degrade low, medium, and high concentrations of MC-LR, making it suitable for MC-LR removal.

**Figure 5:**
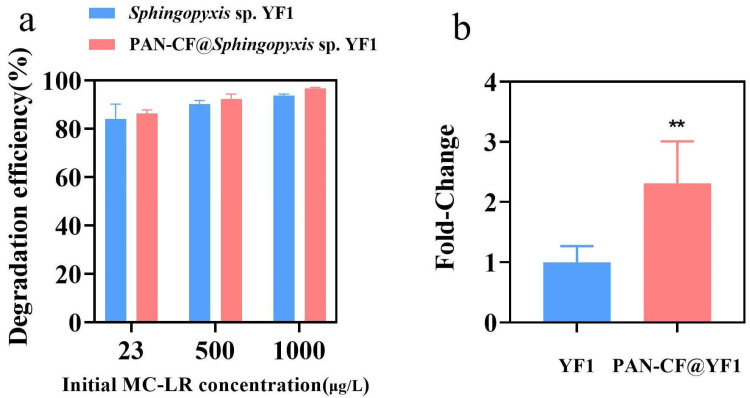
The impact of immobilization on the degradation of MC-LR by *Sphingopyxis* sp. YF1. (**a**) PAN-CF@YF1 and free *Sphingopyxis* sp. YF1 degradation efficiency at 3 h for different concentrations of MC-LR. (**b**) The fold change of *MlrA* in the degradation of MC-LR by *Sphingopyxis* sp. YF1 immobilized on PAN-CF. Symbols: ** *p* < 0.01 vs. CT group.

To further explore the effect of immobilization on *Sphingopyxis* sp. YF1’s degradation of MC-LR, the removal effects of PAN-CF@YF1 and free bacteria on different concentrations of MC-LR at 3 h were studied (Figure 5a), Notably, PAN-CF@YF1 exhibits slightly higher efficiency in eliminating MC-LR compared to free *Sphingopyxis* sp. YF1. Since the adsorption capacity of MC-LR by PAN-CF is limited (Figure S2), the higher removal efficiency of PAN-CF@YF1 than free *Sphingopyxis* sp. YF1 was probably ascribed to the promoted degradation due to immobilization. Previous studies have indicated that the degradation of MC-LR by *Sphingopyxis* sp. YF1 primarily relies on the collective action of enzymes, including *mlrA*, *mlrB, mlrC*, and *PAA*. *MlrA*, as the pivotal enzyme in MC-LR degradation, hydrolyzes the hydrolytic chain between *AddA* and *Arg* in MC-LR, generating a linear product, L-MC-R, with only approximately 1/160th of the original toxicity [47]. The *mlrA* enzyme in *Sphingopyxis* sp. YF1 is regulated by the *mlrA* gene. In this study, we analyzed the expression of the *mlrA* in *Sphingopyxis* sp. Figure 5b shows a 2.48-fold increase (*p* < 0.01) in *mlrA* expression in *Sphingopyxis* sp. YF1 following immobilization on PAN-CF, thereby enhancing *Sphingopyxis* sp. YF1’s capacity to degrade MC-LR. Hai et al. found that microbial enrichment leads to upregulation of gene expression associated with cellular metabolism [48]. Hence, we hypothesize that the disparity in *mlrA* expression between free and immobilization of *Sphingopyxis* sp. YF1 may be attributable to the substantial bacterial attachment on the surface of PAN-CF, resulting in heightened metabolic activity and up-regulate the *mlrA* expression.

### 2.5. Reusability of PAN-CF@YF1

**Figure 6:**
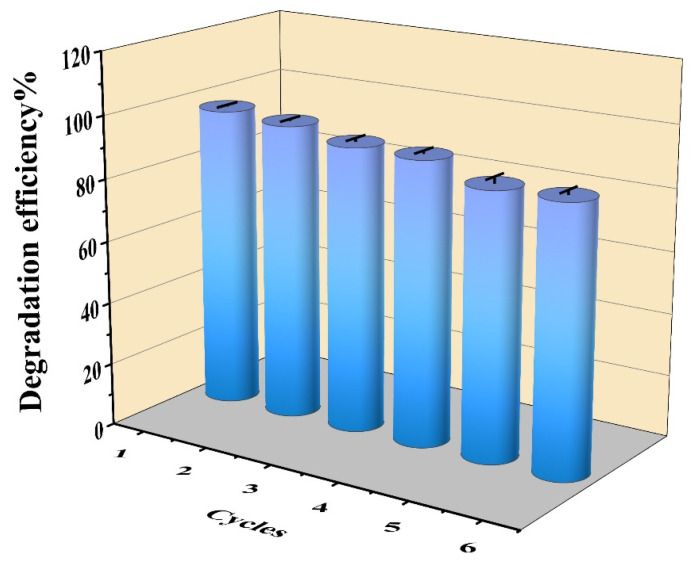
Reusability of PAN-CF@YF1 for the degradation of MC-LR.

Reusability and long-term stability are crucial indicators for evaluating immobilized bacteria in practical applications. These indicators can save time and money by allowing the reuse of bacteria without the need for special treatment, unlike free bacteria [49,50]. In this study, we explored the reusability and recyclability of PAN-CF@YF1. After recycling PAN-CF@YF1, it was utilized to assess its removal capacity for MC-LR (1000 μg/L) within a 3 h timeframe. The degradation ability of PAN-CF@YF1 on MC-LR was examined over six successive cycles, as shown in Figure 6. The removal capacity slightly decreased with each cycle, but remained at 85.75% after the sixth cycle, indicating the outstanding recycling performance of PAN-CF@YF1. Previous studies also showed that the degradation efficiency of immobilized bacteria decreased with an increase in the number of cycles [51]. The decline in this degradation ability may be due to the detachment of bacteria from the bio-material during repeated usage. In a word, PAN-CF@YF1 maintained better stability and sustained relatively high removal efficiency after repeated usage, indicating the potential for practical application.

### 2.6. Degradation of MC-LR by PBBR

**Figure 7:**
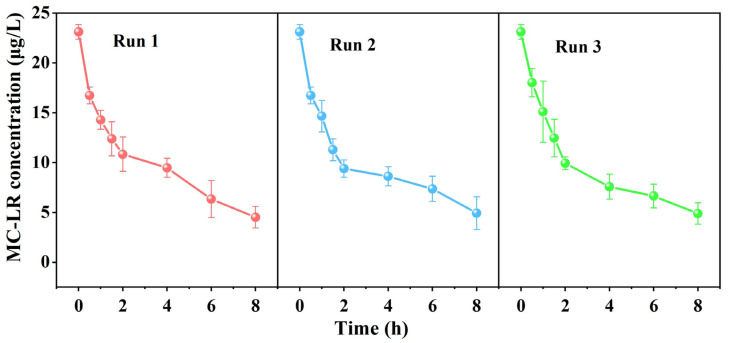
Removal of MC-LR by PBBR for 3 consecutive cycles.

Due to the crucial importance of long-term stability in PBBR’s usage, we investigated its continuous purification capacity for water containing low concentrations of MC-LR. The results of the initial, second, and third cycle experiments are presented in Figure 7. MSM medium containing an initial concentration of 23.82 μg/L of MC-LR was added to the immobilized device, and each cycle lasted for 2 h. The removal efficiencies of MC-LR were 50.21%, 50.25%, and 48.87%, respectively. After 8 h of operation, the removal rates of MC-LR by PBBR were 2.41 μg/L/h, 2.27 μg/L/h, and 2.03 μg/L/h, respectively. These preliminary findings indicate a favorable degradation rate of MC-LR by PBBR. At the end of the operation, the device successfully eliminated 80.43% of MC-LR from the MSM medium. Subsequently, over three experimental cycles, the removal efficiency for MC-LR remained impressively high at 78.83%. These findings unequivocally establish the exceptional operational stability and remarkable capability of this device to efficiently remove MC-LR.

## 3. Conclusions

In this study, a new composite material named PAN-CF@YF1 was effectively formulated for the biodegradation of MC-LR. SEM analysis of PAN-CF@YF1 showed that cells efficiently adhere to the surface of PAN-CF. For optimizing degradation conditions, Box–Behnken design and RSM were utilized. The optimal conditions were determined as follows: a pH of 7.6 for the culture medium, a ratio of 0.038 g of supporting materials per 100 mL of culture media, and an incubation time of 53.4 h. The resultant PAN-CF@YF1 showed high degradation efficiency (86.35–96.61%) for MC-LR with various concentrations. Furthermore, the composite material underwent six cycles to assess its reusability and storage stability. After six cycles, the elimination rate of MC-LR remained at 85.75%. Moreover, PAN-CF@YF1 also showed effective and sustainable performance in the PBBR bioreactor, with a removal rate of 78.83% after three consecutive treatments. As a result, PAN-CF@YF1 exhibits significant potential for the large-scale treatment of MC-LR-contaminated water bodies (e.g., wastewater, lake water), and further work on the removal of MC-LR under different environmental conditions needs to be undertaken to evaluate the scalability and efficiency of the process on a full scale.

## 4. Material and Methods

### 4.1. Materials and Reagents

PAN-CF was acquired from Ningbo Sang Ni Materials Technology Company. Before utilization, PAN-CF was subjected to a 20 min boiling process in ultrapure water at 100 °C and subsequent drying until a stable weight was achieved to eliminate surface impurities. The nutrient broth medium (NB) and mineral salt medium (MSM) were prepared following the methods outlined in a previous study [28]. The Microcystin Plate kit was obtained from Beacon Analytical Systems, Inc (Portland City, ME, USA). The MC-LR used in this experiment was extracted from *M. aeruginosa* FACHB-905, which was procured from the Freshwater Algal Culture Collection at Wuhan Institute of Hydrobiology, Chinese Academy of Sciences. The extraction method of MC-LR aligns with the approach described by Wang [52], resulting in a final concentration of 3600 μg/L of MC-LR.

### 4.2. The MC-LR Degrading Bacterial Strain

*Sphingopyxis* sp. YF1, identified by its GenBank accession number KY491642, was previously isolated from Lake Taihu by our research team [27]. This strain has shown effective capabilities in degrading MC-LR.

### 4.3. Characterization of PAN-CF@YF1

*Sphingopyxis* sp. YF1 was inoculated as a single colony into an MSM medium containing 2000 μg/L of MC-LR. The culture was incubated for 18 h (30 °C, 200 rpm). Subsequently, it was transferred to 50 mL of liquid NB medium and further incubated for 24 h to scale up the culture. Before the experiment, PAN-CF was washed three times with sterile water and subjected to high-temperature and high-pressure sterilization. The sterilized PAN-CF was placed in sterile conical flasks. To each flask containing PAN-CF, 19 mL of sterile liquid NB medium (pH = 7) and 1 mL of activated bacterial suspension (OD_600_ > 1.0) were added. The culture was incubated until *Sphingopyxis* sp. YF1 fully adhered to PAN-CF, forming PAN-CF@YF1.

The morphology of PAN-CF@YF1 was observed using scanning electron microscopy. Before imaging, the sample was immersed in a 2.5% glutaraldehyde solution for 3 h to fix the microbial cells on the surface. Then, the surface cells were dehydrated using a series of ethanol solutions with concentrations of 50%, 70%, 90%, and 100% (each for 10 min). After freeze-drying, the sample was observed using scanning electron microscopy. PAN-CF without fixed-degrading bacteria underwent the same treatment.

### 4.4. The Effect of Different Experimental Conditions on the Immobilization of Sphingopyxis sp. YF1

In order to enhance the immobilization of *Sphingopyxis* sp.YF1 on PAN-CF, adsorption immobilization was performed under varied process conditions, including carrier mass, pH, and immobilization time. During immobilization, 0.01–0.05 g of the material was placed into 19 mL of sterile liquid NB medium (pH = 4–8) containing 1 mL of activated bacterial liquid (OD_600_ > 1.0). The culture was incubated for 16–64 h at 30 °C and 200 rpm. The liquid culture medium in the test tube was discarded, and the fiber surface was washed with sterile PBS to remove the attached bacteria. Then, 10 mL of PBS was added and shaken to release all the immobilized bacteria from PAN-CF. Then, the PBS solution containing bacteria in a sterile centrifuge tube (10 mL) was collected, centrifuged at 6579× *g* for 5 min, and the supernatant was discarded. After centrifuging the bacterial suspension at a speed of 6579× *g* for 5 min, the supernatant was removed, and the bacterial cells were collected and placed in a drying oven. Then, the bacteria were dried until their weight stabilized and then weighed to determine the bacteria’s dry weight. Three parallel samples were set for each group.

### 4.5. Optimization of the Immobilizing Conditions by RSM

RSM is an experimental statistical technique employed to optimize biological processes. It may be used for model creation, factor effect assessment, and optimum conditional factor search for desired results [53,54].

To improve the immobilization of *Sphingopyxis* sp. YF1 on PAN-CF, 17 experiments were performed utilizing a 3 level and 3 factor Box–Behnkens (BBD) design. Table 2 illustrates the levels of each component. The independent variables were pH (A), the ratio of supporting materials (g) to culture media (100 mL) (B), and immobilization time (C). As the output variable, the immobilized *Sphingopyxis* sp. YF1 (g) was used (response). Each design variable was examined at three distinct coded levels (-1, 0, +1). The version 10 of Design-Expert was used for experimental planning and statistical analysis of the results. The significance of the model and the regression coefficient were determined using (analysis of variance) ANOVA (Table 3). Additionally, the determination coefficient was used to assess the accuracy of the polynomial equation [55].

### 4.6. Degradation of MC-LR by PAN-CF@YF1 and Its Reusability

To investigate the degradation capacity of PAN-CF@YF1 towards MC-LR, PAN-CF@YF1 and YF1 were introduced into MSM culture medium containing varied concentrations of MC-LR. To maintain consistency in the number of free and immobilized bacteria on PAN-CF, the immobilized bacteria on PAN-CF were completely shaken off using an NB medium, and their absorbance value was measured (OD_600_). Simultaneously, free bacteria were cultured until reaching the same absorbance value. The MSM culture medium was prepared with three levels of MC-LR concentrations: high (1000 μg/L), medium (500 μg/L), and low (23 μg/L). To ensure experimental consistency, all treatment and control groups were incubated at 30 °C, pH 7, and 200 rpm. Liquid samples were collected at 0.5-hour intervals, and the concentration of MC-LR in each sample was determined using the Microcystin Plate Kit. Each experiment was performed in duplicate to ensure accuracy and reliability.

Furthermore, a reusability study was conducted to evaluate the stability of PAN-CF@YF1. PAN-CF@YF1 was exposed to MSM containing MC-LR (1000 μg/L) to assess its degradation efficiency towards MC-LR over a single trial cycle of 3 h. After the degradation experiment, PAN-CF@YF1 was washed with PBS and then utilized in subsequent degradation experiments. All experiments were conducted under standardized conditions (30 °C, pH 7, and 200 rpm). Samples were collected after 8 h of biodegradation.

### 4.7. qRT-PCR

To examine the changes in mlrA gene expression between the free and immobilized *Sphingopyxis* sp. YF1. RNA from *Sphingopyxis* sp. YF1, whether immobilized on PAN-CF or free in solution, was extracted using the Bacterial RNA Kit (Omega Bio-Tek, Inc., Norcross, GA, USA). RNA concentration and A260/280 ratio were measured using an Ultra-micro ultraviolet-visible spectrophotometer. Subsequently, the RNA was reverse-transcribed to cDNA using a reverse transcription kit. Primers were designed using Primer Premier 5.0 software, and their validity was confirmed by NCBI before being synthesized by Shanghai Biotech, Shanghai, China. The primer sequences used for qPCR are listed in Table S1. The relative mRNA levels were determined using the 2^−ΔΔCt^ method. All experiments were performed in triplicate.

### 4.8. Construction and Operation of Bioreactor for MC-LR Removal

**Figure 8:**
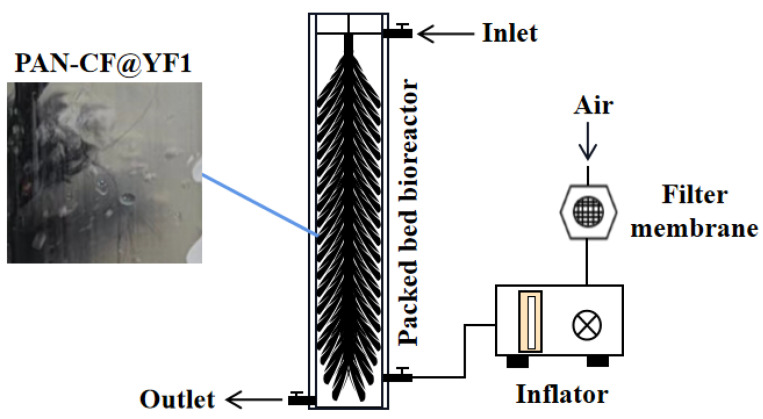
The schematic diagram of MC-LR bioreactor.

In order to apply PAN-CF@YF1 for the treatment of water bodies containing MC-LR, we have designed a PBBR, which exhibits significant advantages in terms of efficiency and portability. The structure of the reactor is shown in Figure 8. The PBBR is made of polymethyl methacrylate (PMMA) with a total volume of approximately 4.7 L. They have a wall thickness of 0.5 cm, an inner diameter of 10 cm, and a height of 60 cm. The top cover with diameter of 11 cm, and the inlet located at the top and the outlet at the bottom. The air inlet is positioned 10 cm above the outlet and equipped with a faucet control switch. The main filling material of the device is PAN-CF, which is boiled in ultrapure water at 100 °C for 20 min, dried until the weight remains unchanged, and then cut into 20 cm segments. Using stainless steel wire as the core material, the fibers are placed around the core and wound clockwise around the stainless-steel wire, forming multiple umbrella-shaped PAN-CF carriers.

Before operating the device, *Sphingopyxis* sp. YF1 is added to the PBBR and incubated. Dry and clean air is continuously supplied through an air pump with a membrane filter (60 mL/min). The liquid culture medium in the PBBR is replaced every 5 days, and an activated *Sphingopyxis* sp. YF1 bacterial solution is added. This process is repeated three times, and the discarded bacterial solution is drained before starting the operation of the device. During the operation, the MSM medium containing MC-LR flows from the inlet into the PBBR. Samples are periodically collected from the outlet to measure the concentration of MC-LR in the MSM medium. Each sample is measured twice to observe the device’s degradation effect on MC-LR. The experiment is repeated three times to assess the stability of the device’s operation.

## Figures and Tables

**Table 1:** ANOVA for response surface quadratic model.

	Sum of Squares	df	Mean Square	F Value	*p*-Value	
Model	375.41	9	41.71	44.22	<0.0001	significant
A	59.59	1	59.59	63.17	<0.0001	
B	27.71	1	27.71	29.38	0.001	
C	81.99	1	81.99	86.93	<0.0001	
AB	4	1	4	4.24	0.0784	
AC	1.56	1	1.56	1.66	0.239	
BC	29.64	1	29.64	31.43	0.0008	
A2	28.25	1	28.25	29.95	0.0009	
B2	72.86	1	72.86	77.24	<0.0001	
C2	52.61	1	52.61	55.77	0.0001	
Residual	6.6	7	0.94			
Lack of Fit	1.6	3	0.53	0.43	0.7446	not significant
Pure Error	5	4	1.25			
Cor Total	382.01	16				

**Table 2:** Levels of parameters and their variation limits.

Factor	Levels		
	−1	0	1
pH (A)	6	7	8
Supporting materials (B)	0.03	0.04	0.05
Time (C)	36	48	60

**Table 3:** Runs of the BBD experiment.

Std.	A	B (g/100 mL)	C (h)	Immobilized Cells (mg/g)
1	1	0	−1	15.00
2	−1	0	1	15.00
3	−1	−1	0	13.33
4	0	−1	−1	15.56
5	1	−1	0	20.00
6	1	1	0	14.67
7	−1	0	−1	10.00
8	0	1	1	18.00
9	1	0	1	22.50
10	0	−1	1	16.67
11	0	1	−1	6.00
12	−1	1	0	12.00
13	0	0	0	22.50
14	0	0	0	22.50
15	0	0	0	22.50
16	0	0	0	21.25
17	0	0	0	20.00

## Data Availability

The data presented in this study are available in this article.

## Supplementary Materials

The following supporting information can be downloaded at: https://www.mdpi.com/article/10.3390/toxins16010020/s1, Text S1: Supplementary information on Box-Behnken design (BBD); Figure S1: The impact of PAN-CF on the growth of *Sphingopyxis* sp. YF1; Figure S2: The adsorption capacity of PAN-CF for MC-LR; Table S1: The primer sequence required for the experiment; Table S2: Comparison of the proposed PAN-CF@YF1 to prior MC-LR degradation studies; References [24,56,57,58,59,60,61] are cited in the supplementary materials.
