# Supplementary material for: Reusable and Practical Biocomposite Based on Sphingopyxis sp. YF1 and Polyacrylonitrile-Based Carbon Fiber for the Efficient Bioremediation of Microcystin-LR-Contaminated Water
Source: Toxins (Basel). 2023 Dec 29;16(1):20. doi: 10.3390/toxins16010020 (PMC10819031; doi:10.3390/toxins16010020)
Supplement: Supplementary file 1 [file toxins-16-00020-s001.zip › toxins-2660348-supplementary.pdf]

Reusable and Practical Biocomposite Based on *Sphingopyxis* sp. YF1 and Polyacrylonitrile-Based Carbon Fiber for the Efficient Bioremediation of Microcystin-LR-Contaminated Water

Tian Ma, Jiajia Zhang, Lili Yang, Shengyu Zhang, Xizi Long, Qingyi Zeng, Zhongyu Li, Xiaoya Ren and Fei Yang

Text S1. Supplementary information on Box-Behnken design (BBD).

Fig. 4a and b are response surfaces and contour map of the relationship between A (pH) and C (time). The figure shows that at immobilization time of 36 h, pH 6 and Supporting materials 0.04 g, immobilized cells reached to 10 mg/g. When pH was increased to 8, while immobilization time was still at 36 h, the immobilized cells increased from 10.00 to 15.00 mg/g. There was a significant increase in the immobilization efficiency of Sphingopyxis sp. YF1 from 10.00 mg/g to 22.50 mg/g when immobilization time and pH changed, from time 60 h, pH 8 to time 48, pH 7. However, the immobilized cells decreased to 15.00 mg/g at time 36 h and pH 8. At low level of pH, the immobilized cells increased to a certain level and then decreased with the increase of immobilization time, while at high level of pH, with the increase of immobilization time, the immobilized cells also increased to a certain level and then decreased. It can be seen from Table 3 that the P values of the AC are higher than 0.05. Therefore, the pH has the same effect on immobilization time, and the interaction between pH and immobilization time is not significant.

Fig. 4c and d show the effect of B (Supporting materials) and C (immobilization time) on the immobilization efficiency of Sphingopyxis sp. YF1 when pH 7 is held constant. From the diagram, it can be observed that the weight of supporting materials plays a vital role in immobilization efficiency under different immobilization time. Conversely, under the different weight of supporting materials, immobilization time also plays a significant role in immobilization efficiency of Sphingopyxis sp. YF1. The immobilization efficiency of Sphingopyxis sp. YF1 increases as immobilization time or the weight of supporting materials increases. At supporting materials of 0.03 g, immobilization time 36 h and pH 7, immobilized cells reached 15.56 mg/g. When immobilization time was increased to 60h, while Supporting materials was still at 0.03 g, the immobilized cells increased from 15.56 to 16.67 mg/g. Supporting materials of 0.05 g, immobilization time 60 h and pH 7, immobilized cells reached 18.00 mg/g. When immobilization time was decreased to 36 h, while supporting materials was still at 0.05 g, the immobilized cells significantly decreased from 18.00 to 6.00 mg/g. The figure shows that at low level of supporting materials, the immobilized cells increased to a certain level and then decreased with the increase of immobilization time, while at high level of Supporting materials, the immobilized cells increased with the increase of immobilization time. It can be seen from Table 1 that F and P values of the BC were 31.43 and lower than 0.05. Therefore, the interaction between immobilization time and Supporting materials is significant.

Fig. 4e and f also demonstrate the effect of pH and Supporting materials on the immobilization efficiency under an immobilization time of 48 h. The figure shows that the immobilization efficiency of Sphingopyxis sp. YF1 gradually increases as pH increases but falls at a point, or gradually increases as the ratio of supporting materials increases but also falls at a point when immobilization time 48 h is held constant. At pH 6, supporting materials 0.03 g and immobilization time 48 h, immobilized cells reached 13.33 mg/g. When pH was increased to 8, while the ratio of supporting materials was still at 0.03 g, the immobilized cells increased from 13.33 to 20.00 mg/g. At pH 6 and 7 and Supporting materials 0.05 g, the immobilized cells was 12.00 and 14.67 mg/g respectively. At low level of pH, the immobilized cells increased to a certain level and then decreased with the increase of supporting materials, while at high level

of pH, with the increase of supporting materials, the immobilized cells also increased to a certain level and then decreased. Therefore, the interaction between pH and supporting materials is not significant.

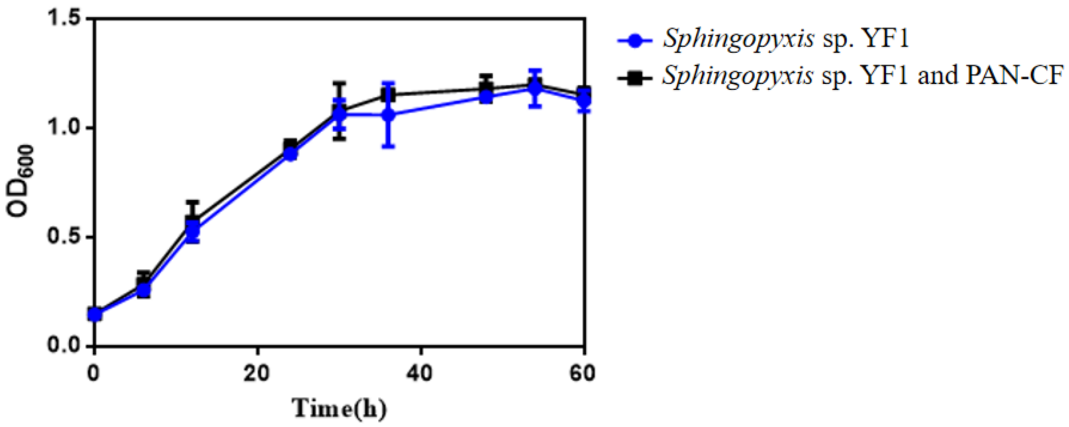

Figure S1 The impact of PAN-CF on the growth of Sphingopyxis sp. YF1.

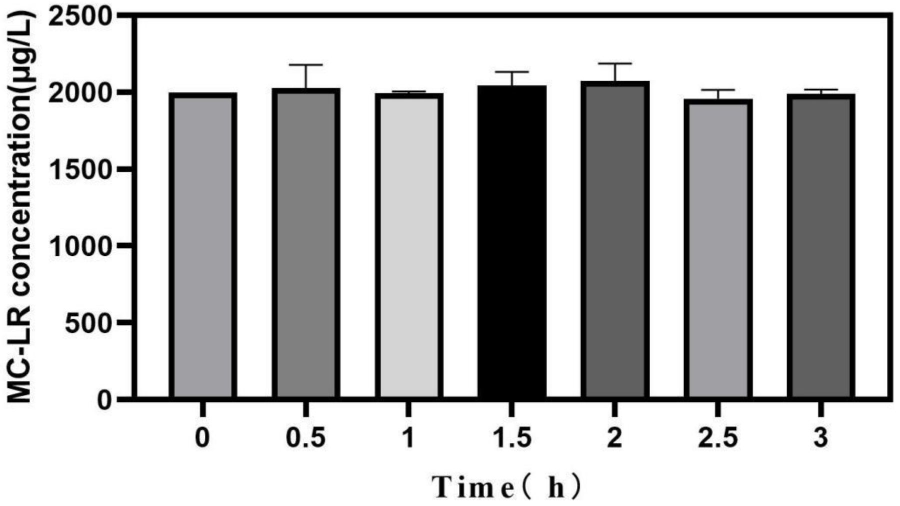

Figure. S2 The adsorption capacity of PAN-CF for MC-LR.

Table. S1. The primer sequence required for the experiment.

Primer Name	Forward Primer	Reverse Primer
YF1 16S rRNA	CCTTACCAGCGTTTGACATCC	CCTTAGAGTGCCCAACTGAATG
mlrA	TCGCGGCTCTTATCGTAACC	AGGCGGAGCGTAGATGAATG

Table S2. Comparison of the proposed PAN-CF@YF1 to prior MC-LR degradation studies.

**Methods**	**Bacteria**	**Supporting materials**	**Initial MC-LR concentration**	**Condition**	**Time**	**Degradation efficiency**	**References**
Free Bacteria	YFMCD4	-	2 μg/mL	pH=7, 30°C (Mineral salt medium)	10 hours	100%	[56]
Free Bacteria	*B. lactis Bb12*	-	0.1 μg/mL	pH=7, 30°C (Mineral salt medium)	24 hours	58.1%	[57]
Free Bacteria	*Sphingomonas isolate NV-3*	-	1 μg/mL	pH=7, 30°C (Mineral salt medium)	24 hours	100%	[58]
Free Bacteria	*Acclimatized-TSFU bacterial community*		0.2 μg/mL	pH=7, 30± 1°C (Mineral salt medium)	15 days	94.35 ± 10.63%	[59]
Immobilization	E.coli BL21-mlrA	Alginate	0.035 μg/mL	pH=7.2, 30°C (fresh water)	-	41.2%	[60]
Immobilization	Sphingopyxis sp. IM-1	RO discarded membranes	2 μg/mL	pH=7, 27°C (Mineral salt medium)	24 hours	100%	[24]
Immobilization	*Novosphingobium sp. KKU25s*	plastic medium	0.025 μg/mL	30 °C, pH 7.2 (fresh synthetic wastewater)	24 hours	100%	[61]
Immobilization	Sphingopyxis sp. YF1	PAN-CF	1 μg/mL	pH=7, 30°C (Mineral salt medium)	3 hours	96.61%	This study
